# Serological Assays Reveal No Evidence of Natural SARS-CoV-2 Infection in US Cattle

**DOI:** 10.3390/microorganisms13030600

**Published:** 2025-03-05

**Authors:** Santhamani Ramasamy, Meysoon Quraishi, Swastidipa Mukherjee, Sonalika Mahajan, Lindsey C. LaBella, Shubhada K. Chothe, Padmaja Jakka, Abhinay Gontu, Sougat Misra, Meera Surendran-Nair, Ruth H. Nissly, Suresh V. Kuchipudi

**Affiliations:** 1 Department of Infectious Diseases and Microbiology, University of Pittsburgh School of Public Health, Pittsburgh, PA 15261, USA; sar465@pitt.edu (S.R.); swm64@pitt.edu (S.M.); lindsey.labella@pitt.edu (L.C.L.); shc319@pitt.edu (S.K.C.); sougat.misra@pitt.edu (S.M.); 2 Center for Vaccine Research, University of Pittsburgh, Pittsburgh, PA 15261, USA; 3 Animal Diagnostic Laboratory, Department of Veterinary and Biomedical Sciences, The Pennsylvania State University, University Park, PA 16802, USA; mjq5073@psu.edu (M.Q.); sonalika.mahajan@icar.gov.in (S.M.); padmaja@psu.edu (P.J.); abhinay@psu.edu (A.G.); meera.snair@antechmail.com (M.S.-N.); rah38@psu.edu (R.H.N.)

**Keywords:** SARS-CoV-2, cattle, antibodies, diagnostics, surveillance

## Abstract

Severe acute respiratory syndrome coronavirus-2 (SARS-CoV-2) continues to pose a significant threat to public health. Notably, SARS-CoV-2 demonstrates the capacity to infect various non-human animal species, including both captive and free-living animals. Earlier experimental studies revealed low susceptibility of domestic cattle (*Bos taurus*) to ancestral B.1 lineage; however, recent experimental findings indicate greater permissiveness of cattle to SARS-CoV-2 Delta variant. While some studies detected evidence of SARS-CoV-2 infection in cattle in Italy, Germany, India, and Nigeria, currently, there is no evidence of SARS-CoV-2 infections in US cattle. We have investigated over 600 samples, including pre-pandemic and pandemic cattle sera collected from Pennsylvania for the presence of SARS-CoV-2 antibodies. Since serological tests have inherent problems of false positives and negatives, we conducted a comprehensive assessment of multiple serological assays. As there are no known SARS-CoV-2 positive cattle serum samples, we used hyperimmune serum raised in cattle with SARS-CoV-2-spike receptor binding domain (RBD) as positive control for the test validation. We found that pseudovirus neutralization assays with a luciferase reporter system can produce false positive results, and care must be taken to interpret serological diagnosis using these assays. We found no serological evidence of natural SARS-CoV-2 infection or transmission among cattle in the US. This study underscores the importance of robust evaluation when employing serological assays for SARS-CoV-2 detection in cattle populations.

## 1. Introduction

The severe acute respiratory syndrome coronavirus-2 (SARS-CoV-2) will remain a threat to public health for the foreseeable future. A remarkable feature of SARS-CoV-2 is the ability to infect many non-human animal species, and natural SARS-CoV-2 infection of multiple captive and free-living animals has been documented [1,2,3,4,5,6]. Receptor binding and membrane fusion are critical steps for coronaviruses to cross the species barrier and establish efficient transmission pathways in new host species. SARS-CoV-2 spike (S) protein mediates virus entry and cell fusion through its direct interaction(s) with the cellular angiotensin-converting enzyme-2 (ACE2) receptor [7,8,9]. The ability of the S protein to bind to ACE-2 receptors is a critical determinant of host susceptibility to SARS-CoV-2 infection.

Comparative and structural analysis of ACE2 receptors in vertebrates predicted that several mammals could be at high risk for SARS-CoV-2 infection [8,9,10]. Based on ACE2 binding to the receptor binding domain (RBD) of the S protein of wildtype B.1 lineage, domestic cattle (Bos taurus) have been predicted to be susceptible to SARS-CoV-2 [2]. Subsequently, experimental studies showed less susceptibility of cattle to ancestral B.1 lineage SARS-CoV-2 infection with low levels of viral replication and limited seroconversion [11,12]. SARS-CoV-2 continues to evolve, resulting in the emergence of mutational variants named after the Greek letters Alpha, Beta, Gamma, Delta, Omicron, and Omicron subvariants, including BA.1 to BA.5 and XBB. The emergence of variants might result in altered host tropism. For example, laboratory mice that were resistant to wildtype SARS-CoV-2 infection were found to be susceptible to alpha and other variants [13,14]. Recently, experimental co-infection of calves found that cattle are more permissive to infection with SARS-CoV-2 Delta than Omicron BA.2 and Wuhan-like isolates [15]. Further, the study also found limited seroconversion and no clear evidence of transmission to sentinel calves [15]. A study in 2022 reported the detection of SARS-CoV-2 antibodies in lactating cows in Italy [16]. Subsequently, a serological survey in Germany found antibody evidence of natural SARS-CoV-2 exposure in cattle [17]. Meanwhile, evidence of SARS-CoV-2 RNA was detected in cattle from India and Nigeria [18,19]. However, these studies showed limited seroconversion in cattle following SARS-CoV-2 infection [11,15,18]. While these studies raise concerns about the potential spillover of recent SARS-CoV-2 variants into cattle, there is currently no evidence of natural SARS-CoV-2 infection in cattle from the US or elsewhere in the world.

Given the experimental evidence indicating low susceptibility, limited seroconversion, and a lack of horizontal transmission of SARS-CoV-2 among cattle, coupled with concerns about the specificity of serological assays, it becomes imperative to thoroughly evaluate various serological methods for detecting SARS-CoV-2-specific antibodies in cattle. Consequently, we conducted a comprehensive assessment of multiple serological assays including pseudovirus neutralization assay (pVNT), surrogate virus neutralization assay (sVNT), in-house developed indirect ELISA and virus neutralization assay (VN) on over 600 cattle serum samples, including pre-pandemic and pandemic sera. Although, we discovered false-positivity in pseudovirus neutralization; cross-verification with other serological tests revealed no serological evidence of natural SARS-CoV-2 infection and transmission of SARS-CoV-2 in cattle in the United States. This study emphasizes the importance of rigorous evaluation when employing serological assays for SARS-CoV-2 detection in cattle populations.

## 2. Materials and Methods

The following materials were obtained through BEI Resources, NIAID, NIH: human embryonic kidney cell line expressing human angiotensin-converting enzyme 2 (HEK-293T-hACE2) (NR-52511); SARS-Related Coronavirus 2 Wuhan-Hu-1 Spike-Pseudotyped Lentiviral Kit V2, (NR-53816). Plasmids encoding spikes of SARS-CoV-2 variants Delta (Cat. No. 172320) and Omicron (Cat. No. 179907) were procured from Addgene, Watertown, MA, USA.

### 2.1. Serum Samples

Cattle serum samples (*n* = 549) submitted to the Animal Diagnostic Laboratory (ADL) at Pennsylvania State University during early 2022 to 2023 for the screening of bovine viral diseases were analyzed in this study for the presence of SARS-CoV-2 antibodies. These serum samples were typically collected by an attending veterinarian as part of their clinical investigation and were subsequently submitted to the ADL. The age of most cattle tested ranged from 2 months to 2 years. Cattle sera (*n* = 49) collected before 2020 were used as pre-pandemic negative controls. As there are no confirmed SARS-CoV-2 positive cattle serum samples, for assay validation, hyperimmune sera (*n* = 3) from cattle immunized with B.1 lineage RBD protein described in our previous study [20] were included as positive controls. All animal care and sample collections were approved and performed in accordance with the guidelines of the Institutional Animal Care and Use Committee at Pennsylvania State University. The Pennsylvania State University Institutional Animal Care and Use Committee (IACUC protocol # PROTO202001506).

### 2.2. Production of SARS-CoV-2 Pseudoviruses

SARS-CoV-2 spike pseudoviruses were produced using the third-generation lentiviral plasmids as described elsewhere [21]. Lentiviral helper plasmids, transfer plasmid encoding luciferase and ZsGreen, and plasmid encoding spike of SARS-CoV-2 variants Delta or Omicron were transfected in HEK 293T cells using Fugene6 reagent (Cat. No. E2691, Promega) following manufacturer’s guidelines. The pseudovirus containing cell culture supernatants were collected after 48 h of transfection, and the infectivity of SARS-CoV-2 pseudoviruses were determined using HEK-293T-hACE2 cells. Briefly, the HEK-293T-hACE2 cells were infected with 10-fold serial dilutions of pseudoviruses in 96 well clear bottom plate (Cat. No. 165306, ThermoScientific, Rochester, NY, USA). At 72 h post infection, RLUs were measured (BioTek Synergy HTX Multi-Mode Microplate Reader, Agilent, Santa Clara, CA, USA) following the addition of BrightGlo luciferase reagent (Cat. No. E2620, Promega, Madison, WI, USA). The dilution of the virus that showed ~10^4^ relative light units (RLU) was used in the pVNT.

### 2.3. SARS-CoV-2 Pseudovirus Neutralization (pVNT) Assay

We employed pVNT to test the presence of SARS-CoV-2 neutralizing antibodies in cattle sera using pseudoviruses containing spike proteins from Delta and Omicron SARS-CoV-2 variants of concern. Briefly, the pseudoviruses were incubated with 1:30 dilutions of sera for an hour at 37 °C and added into 96-well plates containing 1.3 × 10^4^ HEK-293T-hACE2cells. The pseudovirus infectivity was determined at 72 h. The percentage neutralization of pseudoviruses was calculated by normalizing to a virus-only control. Each serum was tested in a single well initially and the samples with percent neutralization of ≥60% were further tested in duplicates at three dilutions (1:30, 1:60, 1:120 and 1:240). A percent neutralization of 60% was further tested in other serological assays. The results were analyzed using GraphPad Prism Software version 9 (San Diego, CA, USA).

### 2.4. SARS-CoV-2 Live Virus Neutralization (VN) Assay

VN assays to determine SARS-CoV-2 neutralizing antibody titers were performed as described earlier [22]. Briefly, Vero E6 cells were seeded onto 96-well plates for 18–24 h at 37 °C with 5% CO_2_. Two-fold serum dilutions mixed with 100 TCID_50_ of SARS-CoV-2 [hCoV-19/USA/PHC658/2021 (lineage B.1.617.2; Delta), and hCoV-19/USA/MD-HP20874/2021 (lineage B.1.1.529; Omicron), BEI Resources, Manassas, VA, USA] and incubated at 37 °C for one hour. The serum-virus mixtures were added to Vero E6 culture and observed for cytopathic effects. The reciprocal of the highest dilution of serum showing no cytopathic effects in at least two of three wells is considered the neutralization titer of the serum.

### 2.5. Bovine Coronavirus Virus Neutralization (VN) Assay

We performed a virus neutralization assay to detect bovine coronavirus specific antibodies following a previously reported procedure [23]. The two-fold serial dilutions of heat inactivated serum were mixed with 100 TCID_50_ of bovine coronavirus strain Mebus and incubated for one hour at 37 °C with 5% CO_2_. The virus and serum mixture were added to MDBK cells, grown in a 96-well microtiter plate, and incubated for 4 to 5 days at 37 °C with 5% CO_2_. The endpoint neutralization titer was designated as the reciprocal of the highest serum dilution, at which the virus infection is inhibited in two of three wells.

### 2.6. SARS-CoV-2 Surrogate Virus Neutralization (sVNT) Assay

We used the widely accepted and extensively validated species-agnostic SARS-CoV-2 antibody detection tool, cPass™ SARS-CoV-2 Neutralization Antibody Detection kit (GenScript, Piscataway, NJ, USA) [24], for testing the cattle serum samples. Briefly, the serum samples were incubated with horse radish peroxidase (HRPO)-conjugated RBD (Delta or Omicron) (Cat. No. Z03614-20 and Cat. No. Z03730-20) and added to the wells coated with human ACE2 protein. The interaction of HRPO-conjugated RBD and ACE2 was determined by measuring the absorbance values after adding the developing solution. The wells showing >30% of inhibition were considered positive for SARS-CoV-2 antibodies.

### 2.7. SARS-CoV-2 RBD-Indirect ELISA

We employed in-house developed indirect ELISA for the detection of antibodies in cattle serum samples [20]. Briefly, SARS-CoV-2 RBD antigens expressed in 293T cells were used as antigen (Cat No. 44240421, Thermofisher, USA) and the plates were incubated with the serum samples diluted in Stabilguard buffer (SG01-1000, Surmodics, MN, USA). Subsequently, anti-bovine IgG peroxidase (Cat # A5295, Sigma-Aldrich, St. Louis, MO, USA) was added to the wells prior to adding 3,3′,5,5′-Tetramethylbenzidine dihydrochloride (Cat # T3405, Sigma-Aldrich, MO, USA) and hydrogen peroxide. OD values were measured at 450 nm using Cytation5 multi-mode reader and samples showing OD values higher than the cut-off values were determined positive for SARS-CoV-2 antibodies.

### 2.8. Statistical Analysis

We have compared the two different cut-off values (60% and 80% inhibition) of SARS-CoV-2 pVNTs with VN. Out of 549 pandemic samples and 49 pre-pandemic samples, 67 samples showed >60% inhibition in SARS-CoV-2 Delta pVNT and 45 samples had >60% inhibition in Omicron pVNT. The specificity of pVNT was calculated using the diagnostic test evaluation calculator, MedCalc web-software: https://www.medcalc.org/calc/diagnostic_test.php (accessed on 21 February 2025).

## 3. Results

### 3.1. Pseudovirus Neutralization Assay Suggests SARS-CoV-2-Specific Antibodies in Cattle Serum

**Figure 1:**
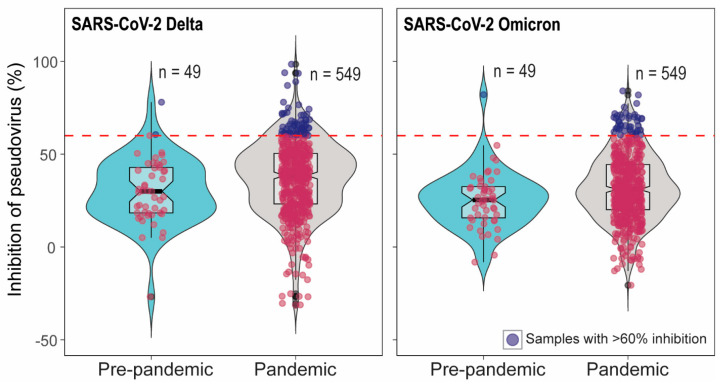
Inhibition percentage of SARS-CoV-2 Delta and Omicron spike pseudoviruses by cattle serum samples in pVNT. In the pVNT, 549 pandemic and 49 pre-pandemic serum samples were tested. The dashed line indicates 60% inhibition. Sixty-five pandemic and two pre-pandemic sera showed >60% inhibition in SARS-CoV-2 Delta pVNT and 44 pandemic sera samples and one pre-pandemic serum showed >60% inhibition in Omicron pVNT. The samples with <60% inhibition are indicated as red dots and the samples with >60% inhibition are indicated as blue dots.

In total 549 pandemic serum samples and 49 pre-pandemic serum samples were tested in SARS-CoV-2 pseudovirus neutralization assays (pVNT). We have previously demonstrated high cross-reactivity of ancestral B.1 RBD-specific hyperimmune serum against pseudovirus expressing pre-Omicron variant spike protein but low cross-reactivity against Omicron pseudovirus [25]. Therefore, pVNT using Delta (pre-Omicron) and Omicron pseudoviruses were performed. Out of 549 pandemic samples, 65 serum samples showed >60% inhibition in pVNT using Delta, and 44 serum samples had >60% inhibition in pVNT using Omicron pseudoviruses. The sixty percent inhibition indicates that the percent inhibition at serum dilution 1:30. However, none of the samples showed >90% inhibition at the 1:30 dilution of serum. Therefore, the 50% neutralization titer lies around 30 which is a very low or inconclusive. Note that 60% inhibition in pVNT is not a positive–negative cut-off in pVNT (Figure 1). Interestingly, 2/49 and 1/49 pre-pandemic serum samples had >60% inhibition in pVNT using Delta and Omicron spike, respectiveley (Figure 1). The quality of serum samples tested was variable, from pale and clear to red or dark brown with debris from blood. To rule out the effect of hemolysis on pVNT results, 33 pale/clear sera and 24 hemolyzed sera were randomly selected for the comparison of percent inhibition in pVNT. Three-fold serial dilutions (1:30 to 1:240) of the samples were tested in pVNT. In pVNT, 33% and 9% of pale/clear and 20% and 16% of hemolyzed samples showed >60% inhibition of RLUs at 1:30 dilution with Delta and Omicron spike pseudoviruses, respectively. Hemolysis and serum quality did not significantly impact whether specimens were above or below 60% inhibition, per two-sided Fisher’s exact test (Delta *p* = 0.56; Omicron *p* = 0.13).

### 3.2. High Percent Inhibition in pVNT Does Not Correspond to Positivity in sVNT, Indirect ELISA and VN

**Figure 2:**
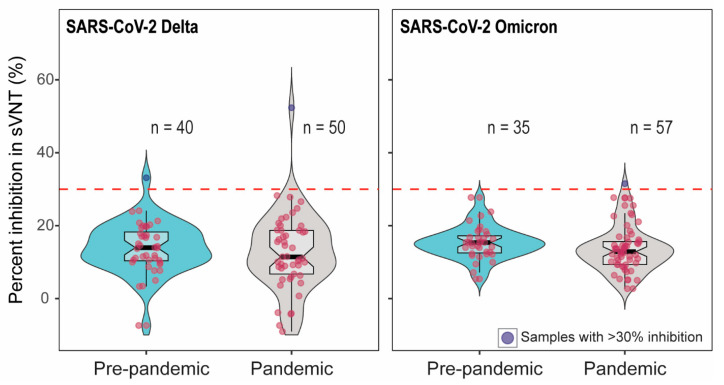
The percent inhibition of cattle sera in Delta and Omicron-RBD based sVNT. The positive–negative threshold stated by the manufacturer is 30%, indicated as dashed line. Two out of 90 and one out of 92 serum samples showed >30% inhibition in Delta and Omicron sVNT, respectively. The red dots indicate the negative samples (below the cut-off) and blue dots depict the positive samples (above the cut-off).

To confirm whether samples with pseudovirus inhibition indicated the presence of SARS-CoV-2-specific antibody, we further tested the serum samples with >60% inhibition in pVNT using two additional assays measuring antibody binding to SARS-CoV-2 RBD. First, we tested sera in surrogate virus neutralization tests (sVNT) using RBD from Delta and Omicron [24,25,26]. Out of 90 samples (52 samples with >60% inhibition and 38 pre-pandemic samples), only two showed the percent inhibition above the cut-off in Delta sVNT. Among these two sera, one was from pre-pandemic samples. Of the 92 samples tested in Omicron sVNT, one sample showed the percent inhibition just above the cut-off (Figure 2). The cattle that showed 55% Delta sVNT inhibition had 71.5% Delta pVNT inhibition; on the other hand, the serum with 33% Delta sVNT inhibition had 4% inhibition in Delta pVNT. The serum with 31% Omicron sVNT inhibition showed 59.5% inhibition in Omicron pVNT.

**Figure 3:**
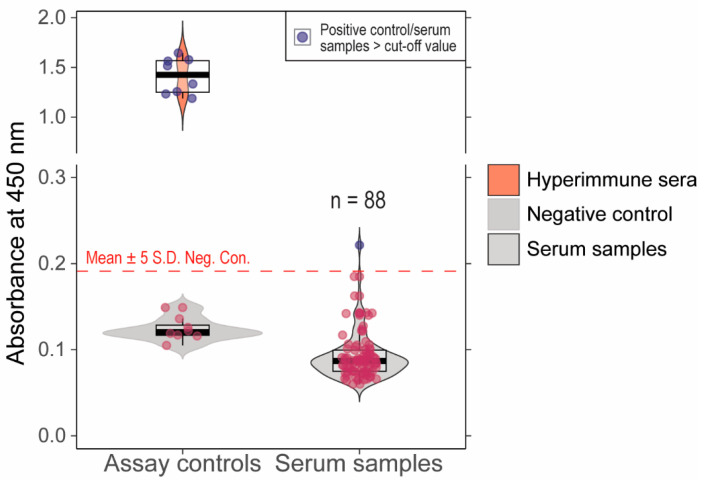
Absorbance values (A_450_ nm) for cattle serum samples tested in in-house developed indirect ELISA. The cut-off for the positive vs. negative samples is Mean + 5S.D shown as dashed line. One of the serum samples had absorbance values higher than the cut-off. The red dots indicate the negative samples (below the cut-off) and blue dots depict the positive samples (above the cut-off).

We previously validated an ancestral B.1 lineage RBD indirect ELISA assay with 100% sensitivity and specificity compared to a live virus neutralization assay [20]. When serum samples (*n* = 88) that showed >60% inhibition in pVNT were tested in this assay, one sample showed absorbance above the determined cut-off and 87 samples had absorbance below the cut-off (Figure 3). Further, the samples that showed >30% inhibition in Delta (*n* = 2) and Omicron (*n* = 1) sVNT were negative in the indirect ELISA assay. The serum (*n* = 1) that was positive in indirect ELISA had 45% inhibition in Delta pVNT. The serum samples with positivity in at least one of the serological assays are indicated in Table 1. The serum samples with >60% inhibition in pVNT and pre-pandemic samples were tested in live virus neutralization assays; none of the samples showed neutralization at 1:20 dilution.

### 3.3. Diagnostic Specificity of pVNT and the Cut-Off Determination

We have employed the 60, 80, and 90% inhibition values as positive–negative thresholds to determine the specificity of the pVNT as compared to the gold standard live VNT (Table 2). At 60% cut-off, the Delta and Omicron pVNTs showed 88.79% and 92.47% specificity, respectively. Increasing the cut-off to 80% inhibition, resulted in >99% specificity in both Delta and Omicron pVNTs, suggesting the 80% cut-off would reduce the false positive results (Table 2). Due to the lack of SARS-CoV-2 VN positive serum samples, we were unable to determine the diagnostic sensitivity of the pVNT.

### 3.4. SARS-CoV-2 Specific Cattle Antibodies Are Not Cross-Reactive to Bovine Coronavirus (BCoV)

BCoV, like SARS-CoV-2, is a member of the Betacoronavirus genus. BCoV is widespread in cattle populations and causes respiratory and enteric infections. Vaccination against BCoV is a common management strategy in the US. To understand if our observed SARS-CoV-2 pseudovirus inhibition could be due to cross-reactive bovine coronavirus antibodies, we tested a subset of cattle serum samples in BCoV live virus neutralization assays. We analyzed three hyperimmune sera, five serum samples that showed >60% inhibition in pVNT, 10 samples that showed <60% inhibition in pVNT, and three prepandemic serum samples (a serum showed >60% inhibition in Omicron pVNT). One hyperimmune serum, two pandemic serum samples with >60% inhibition, and four pandemic serum samples with <60% inhibition in pVNT showed neutralization of BCoV (Table 3). The majority of samples (60%) showed no neutralization of BCoV regardless of their SARS-CoV-2 pVNT status. These results indicate that percent inhibition in the SARS-CoV-2 pVNT assay does not correlate with BCoV neutralization. Notably, SARS-CoV-2 Wuhan RBD hyperimmune sera that had >90% inhibition in the pVNT assay failed to exhibit cross-neutralization against BCoV (Table 3).

## 4. Discussion

Cross-species transmission can occur when viruses contact the potential new hosts [27]. Abundant, sustained, and protracted human-to-human transmission of SARS-CoV-2 promotes the risk of spillover to susceptible animal species. Natural infection and circulation of SARS-CoV-2 has been well-established in white-tailed deer, the most abundant large mammal species in the US [28,29,30,31,32]. Shared home ranges enhance the potential for spillover of SARS-CoV-2 from white tailed-deer to cattle. It is well-established that bacterial and viral pathogens can be transmitted between deer and cattle due to the overlap of deer home ranges with cattle pastures. Mycobacterium tuberculosis and bovine viral diarrhea viruses are thought to persist through bidirectional transmission between cattle and deer [33,34], and transmission between species has been documented for bovine coronavirus [35]. Transmission of SARS-CoV-2 within the human population occurs through aerosols, droplets, and fomites, possibly through either direct or indirect contacts [36,37]. The potential for transmission of SARS-CoV-2 to livestock from humans and wildlife through similar routes is high.

Though an experimental study suggested cattle are poorly permissive to infection with SARS-CoV-2 [11,12], a recent study discovered that cattle are more permissive to infection with SARS-CoV-2 Delta than Omicron BA.2 [15]. Further, the study also found limited seroconversion and no clear evidence of transmission to sentinel calves [15]. Serological studies from Italy [16] and Germany [17] found antibody evidence of natural SARS-CoV-2 exposure of cattle. Serological assays have advantages over antigen detection methods for surveillance due to the larger window of detection of antibodies than the viral antigens. However, serological tests show variable sensitivities [38,39], and false-positive serology test results have been reported in COVID-19 [40,41]; therefore, it is crucial to compare various serological testing methods in a given host species for serological determination of SARS-CoV-2 infection. Further, serological testing may not capture active infections or cases with minimal seroconversion.

We investigated antibody presence in cattle using an easily adaptable pVNT permitting detection of antibodies to ancient and contemporary SARS-CoV-2 spike. With stringent testing using multiple serological and neutralization assays, all the US cattle serum (*n* = 598) were negative for SARS-CoV-2 antibodies. Notably, one serum sample showed borderline positive results in both pVNT and sVNT using Omicron antigen. However, the sample was negative in SARS-CoV-2 VN.

Although pVNTs yield comparable neutralization titers as that of VN for detecting the SARS-CoV-2 antibodies [42,43,44], their use as a diagnostic tool could be limited due to the highly sensitive luciferase reporter. pVNTs are widely used to determine the SARS-CoV-2 variant specific neutralization titer [25]. We found that high percent inhibition of pseudovirus in 1:30 dilution did not predict antibody detection ability in other methods, including sVNT. When pVNTs are repurposed to use for diagnosis using a single serum dilution, several factors may contribute to false positive results. In general, when the serum has a good neutralizing antibody titer to SARS-CoV-2, the percent inhibition in pVNTs are ~100% in several two-fold serial dilutions, up to 1:120 dilution of cat serum [25] and white-tailed deer [26]. Meanwhile, the cattle serum samples that were tested in pVNT showed inhibition from 0 to 80% and a few sera had >80% inhibition at 1:30 dilution. Here, the reduction in pseudovirus readout could be due to cytotoxicity at 1:30 dilution, as the reduced cell growth could result in less luminescence. A way to prevent false positive results due to less cell growth is by quantitating protein concentrations. The determination of appropriate cut-off could increase the specificity of the pVNT. However, it requires VN positive (true positive) serum samples to achieve reliable cut-off without compromising the diagnostic sensitivity and specificity.

The GenScript c-Pass sVNTs are widely used for sero-surveillance in humans that employ the 30% inhibition as a cut-off [24]. SARS-CoV-2 Delta-RBD based sVNT showed 99.93% specificity and 95–100% sensitivity detecting the antibodies in humans [24]. Being a species agnostic test, sVNT has been evaluated for the antibody detection in different species including white-tailed deer, cat, hamster [45]. We have used 30% cut-off for cattle sera analysis; however, a recent study recommended the cut-off of 43% and 51% using limited numbers of pre-pandemic cattle and horse sera suggesting the cut-off of 30% may yield false positives [46]. Therefore, the Delta and Omicron-sVNT positive samples (*n* = 3) in this study could be due to incorrect cut-off, which is supported by two of the pre-pandemic samples showing more than 30% inhibition in Delta-sVNT. In indirect ELISA, we have established the cut-off (OD + 5SD) using 40 pre-pandemic serum samples [20]; however, this cut-off was not evaluated with the clinical samples from natural SARS-CoV-2 infection due to lack of positive samples. Therefore, we assumed the possibility of false-positive results in the indirect ELISA and tested all the samples with >60% inhibition in pVNT using VN. However, none of the serum samples with >60% inhibition in Delta and Omicron-pVNTs showed the neutralization of SARS-CoV-2 in VN. Although, VN assays are gold-standard comparative tests for antibody detection, they require BSL-3 facility [47]. In most cases, BSL-3 laboratories are shared facilities for multiple users and require use of expensive personnel protective equipment; therefore, it is not feasible to test large number of samples for the diagnostic purposes using SARS-CoV-2 VN.

A key limitation of our study is that sampling was restricted to cattle aged 2 months to 2 years in Pennsylvania, a state on the U.S. East Coast. As a result, our findings may not fully apply to older cattle or those with underlying health conditions.

While our samples were specific to Pennsylvania, we anticipate similar transmission trends across the United States, given the consistent dairy and beef cattle practices and the shared characteristics of wildlife habitats. With its abundant white-tailed deer population, Pennsylvania presents a unique ecological setting where widespread SARS-CoV-2 circulation in deer could potentially facilitate virus transmission from deer to cattle.

However, a large-scale, continuous monitoring effort across the U.S. is crucial to gain deeper insights into the spillover potential of recent SARS-CoV-2 variants in cattle populations. Such comprehensive surveillance would strengthen our understanding of emerging risks and support proactive measures to safeguard animal and public health.

Considering the wide range of non-human mammalian species that are susceptible to SARS-CoV-2, it is entirely possible that new variants capable of infecting cattle could emerge. Unfortunately, current surveillance efforts in domestic and wild animal populations remain inadequate, making it challenging to accurately assess the risk of spillover events into animal species. Our study highlights the need for caution when interpreting serological diagnoses, as existing assays may lack the specificity needed for high-risk animal populations. Consequently, there is an urgent need to develop reliable serological assays that are specifically designed to detect SARS-CoV-2 antibodies in animals, particularly those at greater risk of exposure. This work underscores the importance of rigorous evaluation protocols to ensure the accuracy of serological testing, ultimately strengthening our ability to monitor and manage potential zoonotic transmission events and protect both animal and public health.

## Figures and Tables

**Table 1:** Results demonstrating “positivity” in at least one SARS-CoV-2 serological assay.

Serum ID	Delta pVNT	Omicron pVNT *	Delta sVNT *	Omicron sVNT *	iELISA *	VN *
P2231751-2	71%	84%	52%	Neg	Neg	<20
P2002039-2	4%	20%	33%	Neg	Neg	<20
2C	49%	59.5%	Neg	31%	Neg	<20
P2214655-57	58.3%	64%	Not done	Neg	Pos	<20

* pVNT: Pseudovirus neutralization test, sVNT: Surrogate virus neutralization test, iELISA: Indirect ELISA, VN: Live virus neutralization assay.

**Table 2:** Diagnostic specificity and false positive rate of pVNT at different cut-offs.

		VN	pVNT			
Assay Target	Total Samples	Positive Samples	Cut-Off (% Inhibition)	Positive Samples	False Positives	Specificity (95% CI)
SARS-CoV-2 Delta	598	0	60%	67	11.20	88.79% (85.99% to 91.21%)
			80%	5	0.83	99.16% (98.06% to 99.73%)
			90%	3	0.50	99.50% (98.54% to 99.90%)
SARS-CoV-2 Omicron	598	0	60%	45	8.1	92.47% (90.06% to 94.46%)
			80%	3	0.54	99.50% (98.54% to 99.90%)
			90%	0	0	100.00% (99.39% to 100.00%)

**Table 3:** Determination of Bovine coronavirus neutralization titer of pre-pandemic (*n* = 2), pandemic (*n* = 15) and hyperimmune (*n* = 3) serum samples.

Type of Samples	BCoV Neutralization			
	Number of Samples	Positives	% Positive	Neutralization Titer
RBD hyperimmune	3	1	33%	1280, <20, <20
>60% inhibition	5	2	40%	160, 320, other samples <20.
<60% inhibition	10	4	40%	20, 80, 20, 20, other samples <20.
Pre-pandemic samples	2	0	0%	All the samples <20%

## Data Availability

Dataset available on request from the authors: The raw data supporting the conclusions of this article will be made available by the authors on request.
